# Polyamidoamines Derived from Natural α-Amino Acids as Effective Flame Retardants for Cotton

**DOI:** 10.3390/polym13213714

**Published:** 2021-10-28

**Authors:** Alessandro Beduini, Federico Carosio, Paolo Ferruti, Elisabetta Ranucci, Jenny Alongi

**Affiliations:** 1 Dipartimento di Chimica, Università degli Studi di Milano, via C. Golgi 19, 20133 Milano, Italy; alessandro.beduini@unimi.it (A.B.); paolo.ferruti@unimi.it (P.F.); elisabetta.ranucci@unimi.it (E.R.); 2 Dipartimento di Scienza Applicata e Tecnologia, Politecnico di Torino, Alessandria Campus, viale T. Michel, 15121 Alessandria, Italy; federico.carosio@polito.it

**Keywords:** linear polyamidoamines, α-amino acids, intumescent flame retardants, functional coatings, cotton

## Abstract

In this paper, bioinspired polyamidoamines (PAAs) were synthesized from *N,N*′-methylenebisacrylamide and nine natural α-amino acids: *L*-alanine, *L*-valine, *L*-leucine (M-LEU), *L*-histidine, *L*-serine, *L*-asparagine, *L*-glutamine (M-GLN), *L*-aspartic acid and *L*-glutamic acid (M-GLU) and their performance as flame retardants (FRs) for cotton were determined. The aim was to ascertain if the ability to protect cotton from fire by the process of intumescing, previously found for the glycine-derived M-GLY, was a general feature of α-amino acid-derived PAAs. None of the PAAs ignited by flame impingement, apart from M-LEU, which burned for a few seconds leaving 93% of residue. All of them formed carbon- and oxygen-rich, porous chars with a graphitic structure in the air at 350 °C, as revealed by X-ray photoelectron spectroscopy. All samples were tested as FRs for cotton by horizontal flame spread tests. At a 5% add-on, M-GLU and M-GLN extinguished the flame. The same results were obtained with all the other PAAs at a 7% add-on. The α-amino acid residues influenced the FR performance. The most effective were those that, by heating, were most suitable for producing thermally stable cyclic aromatic structures. All PAA-treated cotton samples, even when burning, left significant residues, which, according to scanning electron microscopy analysis, maintained the original cotton texture.

## 1. Introduction

The aza-Michael polyaddition of *prim*- or bis-*sec*-amines with bisacrylamides leads to a family of multifunctional polymers named polyamidoamines (PAAs) [1,2,3,4]. The polymerization reaction is favored by protic solvents and is highly specific. Only a few chemical functions other than amines, such as thiols and phosphines, can interfere with the polymerization reaction. Therefore, many other chemical functions can be introduced in PAAs as side substituents and this whole class is highly versatile. Interestingly, PAAs can be designed to be biocompatible; therefore, they have been extensively studied for biotechnological applications [5,6].

Natural α-amino acids can also be employed as comonomers in PAA synthesis, giving rise to a scion of PAAs named polyamidoaminoacids (PAACs) [7], which, incidentally, are easily amenable to controlled synthesis [8]. Some α-amino acid-derived PAAs, particularly those deriving from the polyaddition of *N,N*′-methylenebisacrylamide (MBA) with glycine and *L*-arginine, were recently shown to be non-flammable by applying a propane flame. In fact, contact with the flame induced a superficial intumescence leaving the inner layers white and apparently unaltered [9]. The same PAAs proved endowed with potential as intumescent flame retardants (FRs) for cotton. It has long been recognized that intumescence plays a key role in providing FR properties [10]. Subsequently, it was found that the introduction in the polymer chain of disulphide groups, for instance by using *L*-cystine as monomer [11,12] or comonomer [13], dramatically increased the FR properties. In particular, cotton fabrics treated with disulphide-containing PAAs did extinguish the flame both in horizontal flame spread tests (HFSTs) and more severe, vertical flame spread tests (VFSTs) in the add-on range from 8–12%.

It remained to ascertain whether the ability to intumesce on contact with a flame and act as a FR was a general feature of this family of PAAs. Therefore, a library of PAAs deriving from MBA and nine different natural α-amino acids was synthesized and studied for their FR performance and the influence of the amino acid α-substituent. Specifically, the α-amino acids considered were *L*-alanine, *L*-leucine, and *L*-valine, bearing an increasingly large hydrophobic side chain; *L*-serine, *L*-asparagine, and L-glutamine bearing a neutral hydrophilic side chain; *L*-aspartic acid and *L*-glutamic acid bearing acidic side substituents, and *L*-histidine bearing a basic heterocyclic side substituent. As a benchmark, the glycine-derived PAA was chosen.

It may be finally observed that the synthesis of α-amino acid-based PAAs does not employ organic solvents nor added metal catalysts. Moreover, these polymers do not contain elements, such as bromine or phosphorous, liable to develop obnoxious substances upon heating and the experiments are carried out in water at room temperature, in line with the present trend of developing sustainable solutions for FRs for polymeric materials [14,15,16].

## 2. Materials and Methods

### 2.1. Materials

Glycine (coded as GLY, 98%), *L*-alanine (ALA, 99%), *L*-valine (VAL, >99%), *L*-leucine (LEU, >98%), *L*-histidine (HIS, >99%), *L*-serine (SER, 99%), *L*-asparagine (ASN, >98%), *L*-glutamine (GLN, 99%), *L*-aspartic acid (ASP, >99%), *L*-glutamic acid monohydrate (GLU, >98%), *N,N*′-methylenebisacrylamide (MBA, 99%), lithium hydroxide monohydrate (98%), and HCl 1M (aqueous solution), were supplied by Sigma-Aldrich (Milano, Italy) and used as received.

Cotton (COT) with an area density of 240 gm^−2^ was purchased from Fratelli Ballesio S.r.l. (Torino, Italy).

### 2.2. Methods

The chemical structure of PAAs was assessed by ^1^H Nuclear Magnetic Resonance (NMR), collecting spectra in D_2_O at pH 4.0 and 25 °C using a Bruker Advance DPX-400 NMR spectrometer (Milano, Italy) operating at 400.13 MHz. PAAs and PAA-treated cotton fabrics were analyzed by Attenuated Total Reflectance (ATR) Fourier Transform Infrared Spectroscopy (FT-IR). FT-IR/ATR spectra were recorded at room temperature, in 4000–500 cm^−1^ range, with 32 scans and 4 cm^−1^ resolution, using a Perkin-Elmer Frontier FT-IR/FIR spectrophotometer (Milano, Italy), equipped with a diamond crystal characterized by a penetration depth of 1.66 µm.

Size exclusion chromatography (SEC) traces were obtained for all polymers with Toso-HaasTSK-gel G4000 PW and TSK-gel G3000 PW columns connected in series, using a Waters model 515 HPLC pump (Milano, Italy) equipped with a Knauer autosampler 3800 (Knauer, Bologna, Italy), a light scattering (670 nm), a viscometer Viscotek 270 dual detector (Malvern, Roma, Italy), and a refractive index detector (Model 2410, Waters, Milano, Italy). The mobile phase was a 0.1 M Tris buffer (pH 8.00 ± 0.05) solution with 0.2 M sodium chloride (sample concentration: 20 mg mL^−1^; flow rate: 1 mL min^−1^; injection volume: 20 μL; loop size: 20 μL; column dimensions: 300 mm × 7.5 mm). The instrument optical constants were determined using PEO 24 kDa as a narrow standard. Before analysis, each sample was filtered through a 0.2 μm Whatman^TM^ syringe filter (Maidstone, UK).

The surface morphologies of untreated and PAA-treated cotton fabrics were analyzed by an EVO 15 equipped with a ULTIM MAX 40 probe scanning electron microscope (SEM) manufactured by Zeiss (Ramsey, NJ, USA) and operating at 8.5 mm working distance, under 5 kV beam voltage. A fabric piece (5 mm × 5 mm) was fixed to conductive carbon adhesive tape and then gold-metallized.

Thermogravimetric analyses (TGA) of PAAs and PAA-treated cotton fabrics were performed in inert (nitrogen) and oxidative (air) atmosphere on a TAQ500 thermogravimetric balance from 50 to 800 °C (heating rate 10 °C min^−1^); samples (5 mg) were placed in open alumina crucibles, in either inert or oxidative atmospheres under 20 mL min^−1^ gas flow.

PAA residues heated at 350 °C in an oven were analyzed using an X-ray photoelectron spectrometer (XPS) equipped with an Al K radiation monochromatic source (1486.6 eV) and manufactured by Surface Science Instruments—Biolin Scientific UK (Manchester, UK).

### 2.3. Synthesis of PAAs

Synthesis of M-GLY 

M-GLY was synthesized as already reported [9]. In brief, MBA (15.40 g; 0.10 mol), glycine (7.50 g; 0.10 mol), and lithium hydroxide monohydrate (4.20 g; 0.10 mol) were dissolved in water (35 mL). The reaction mixture was heated to 40–45 °C until the complete dissolution of MBA and then left for 5 days at 25 °C in the dark under nitrogen. It was then diluted to 100 mL with water, the pH was adjusted to 4.5 with 37% hydrochloric acid. The final product was retrieved by freeze-drying the retained portion. The yield was nearly quantitative. 

All other PAAs were obtained as reported from M-GLY, using the amounts of reagents reported in Table 1.

### 2.4. Treatment of Cotton Fabrics with PAAs

Strips of cotton fabric of size 30 mm × 60 mm were dried by heating at 100 °C for 2 min and then weighed. Subsequently, they were impregnated twice with 5 mL aqueous solutions of PAAs of suitable concentration, and were dried for 3 min at 100 °C after each deposition. The total dry, solid add-ons (*Add-on*, wt.%) were determined by weighing each sample before (*W_i_*) and after drying following impregnation (*W_f_*). The add-ons were calculated according to Equation (1):

(1) $$Add-on=\frac{W_{f}-W_{i}}{W_{i}}\times 100$$

The concentrations of the impregnating PAA solutions and the final add-ons were: 2.5 wt.% for 5% add-on and 3.5 wt.% for 7% add-on.

Treated cotton fabrics were coded with the prefix COT/ and, subsequently, the name of homopolymer used, COT/M-ALA, coded the cotton sample treated with the M-ALA homopolymer.

### 2.5. Combustion Tests of PAAs and PAA-Treated Cotton Fabrics

PAA ignitability was tested with the application of a 20 ± 5 mm long butane flame, directly in contact with the powdered polymer, for 10 s. The propane flame contacted the surface of the PAA sample at an angle of 45°. All tests were conducted in triplicate and residual mass fraction (RMF, %) was assessed.

In horizontal flame spread tests (HFSTs), a 20 ± 5 mm long butane flame was applied for 3 s to the short side of the COT/PAA samples; the specimens were positioned in a metallic frame tilted at an angle of 45° along their longer axis and then ignited. All combustion tests were tripled and the total combustion time (s) and residual mass fraction (RMF, %) were assessed.

The resistance to a 35 kW m^−2^ irradiative heat flux of square fabric samples (100 mm × 100 mm) was investigated using an oxygen-consuming cone calorimeter (Noselab ATS advanced, Milan, Italy). Measurements were carried out in horizontal configuration following a procedure previously reported [17], optimized on the basis of the ISO5660 standard [18]. Parameters such as the time to ignition (TTI, s), peak of heat release rate (pkHRR, kW m^−2^), total heat release (THR, MJ m^−2^), and residual mass fraction (RMF, wt.%) were determined. Carbon monoxide (CO) and carbon dioxide (CO_2_) yields, expressed in kg^−1^, were also determined. In order to establish the FR efficiency of PAAs, the Fire Performance Index (FPI), that is, the TTI to pkHRR ratio, was calculated: the higher the FPI, the more efficient the FR system [19]. Prior to the combustion tests, all specimens were conditioned to constant weight at 23 ± 1 °C for 48 h at 50% relative humidity in a climatic chamber. Each experiment was performed in triplicate and the mean standard deviation calculated.

## 3. Results and Discussion

### 3.1. Synthesis of PAAs

**Scheme 1:**
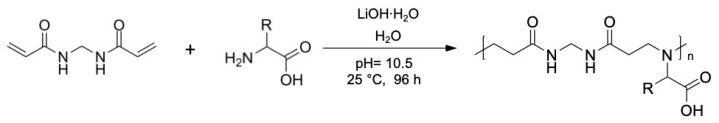
Synthesis of PAAs.

A library of nine PAAs was prepared by the aza-Michael polyaddition of *N,N*′-methylenebisacrylamide (MBA) with L-alanine (M-ALA), L-valine (M-VAL), L-leucine (M-LEU), L-histidine (M-HIS), L-serine (M-SER), L-asparagine (M-ASN), L-glutamine (M-GLN), L-aspartic acid (M-ASP), and L-glutamic acid (M-GLU), as previously reported [9]. In addition, M-GLY, already studied as FR for cotton [9], was prepared from MBA and glycine to serve as a benchmark for assessing any intumescent behavior and FR ability for cotton of all PAAs considered. Briefly, all samples were prepared in a single step in water at pH 10.5 and a 40–45 wt.% concentration (Scheme 1). The reaction mixtures gradually became homogeneous and, after being left to rest under occasional stirring at 25 °C for 5 days, were lyophilized. All products were used with no further purification as, after having been purified by ultrafiltration, they showed no different FR performance compared to the raw products.

The structures of all PAAs considered, reported in Table 2, were confirmed by ^1^H-NMR and ATR/FT-IR spectroscopies (Figures S1–S11, respectively). It was found, particularly in the case of M-ASN, M-HIS and M-GLU, that the NMR spectra showed significant quantities of residual double bonds, corresponding to an average molecular weight, ${\bar{M}}_{n},$ in the range from 3000 to 4000. The reaction yields reached a completeness by carrying out the reaction at 50 °C at a concentration of 60 wt.%. However, in these cases, the products gave evidence of containing from 10 to 30 wt.% cross-linked by-products due to thermally induced, radical polymerization. Therefore, this synthetic method was abandoned. The ${\bar{M}}_{n}$ of the remaining PAAs, as determined by size exclusion chromatography (SEC), ranged from 7500 to 10,000, with polydispersity index (PD) about 1.4.

### 3.2. Thermal Stability of PAAs

**Figure 1:**
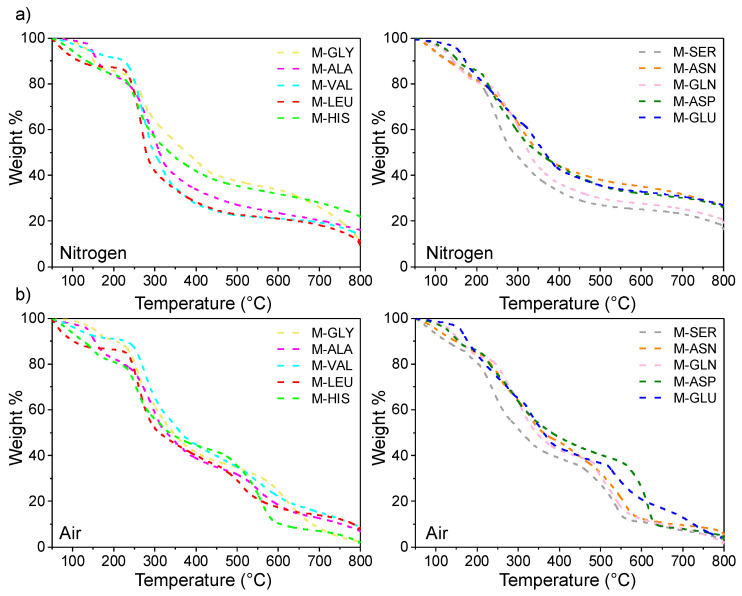
TG thermograms of α-amino acid-derived PAAs in nitrogen (**a**) and air (**b**).

The TG thermograms of all the α-amino acid-derived PAAs that were considered, obtained in nitrogen and air between 50 and 800 °C, are shown in Figure 1. The related T_onset10%_, the onset decomposition temperature at a 10% weight loss; T_max_, the temperature at maximum weight loss rate, and RMF, the residual mass fraction measured at 800 °C, are reported in Table 3. As previously reported for several PAAs [9], and in the case of the PAAs considered in this work, the TG curves in air were very similar to those in nitrogen, up to 350 °C. At higher temperatures, however, the two sets of the curves diverged, as in the air the char formed during the previous decomposition phase, decomposing into volatile oxidation products. Overall, the T_onset10%_ in nitrogen ranged from 116 to 157 °C and those in air ranged from 102 to 159 °C. All PAAs showed in nitrogen exhibited a single main decomposition step with T_max_ ranging from 230 to 315 °C, whereas in air they exhibited two main decomposition steps, occurring between 225 and 270 °C (T_max1_), and between 500 and 616 °C (T_max2_).

It should be noted that all the PAAs considered are remarkably stable. That said, their thermal stability in nitrogen and air can be ranked by comparing the RMF_800_ and T_max2_ values, respectively. By adopting these criteria, it would appear that the stability ranking in nitrogen is: M-ASP, M-ASN, M-GLU > M-GLN ≈ M-HIS > M-SER > M-ALA > M-VAL > M-GLY > M-LEU, and the stability ranking in air is: M-ASP > M-GLY > M-ASN ≈ M-HIS > M-ALA > M-VAL, M-SER > M-LEU > M-GLN. Interestingly, M-ASP, with two lateral carboxylic groups and the shortest methylene chain, turns to be the most thermally stable, whereas M-LEU, with the longest lateral alkyl group, is almost the least stable.

**Figure 2:**
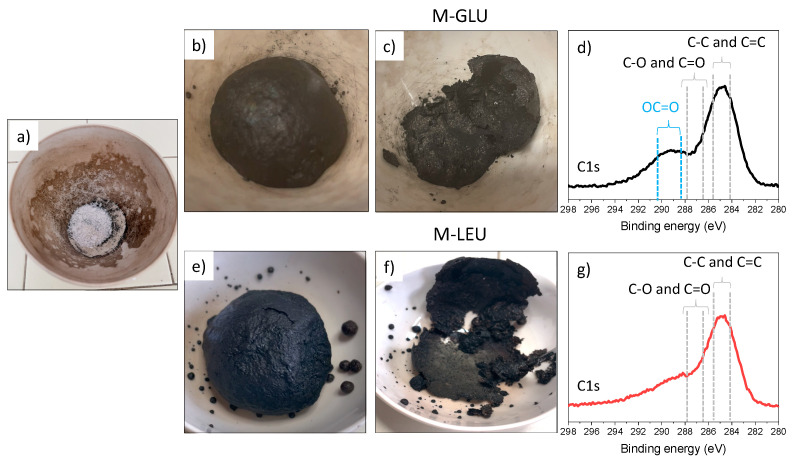
Intumescent behavior of M-GLU and M-LEU: (**a**) typical PAA powder sample before heat treatment; (**b**,**e**) expanded chars immediately after heating to 350 °C; (**c**,**f**) cut surfaces of the char samples; (**d**,**g**) XPS spectra of chars.

As already reported elsewhere [9], the PAAs are derived from α-amino acids intumesce in the air during the second decomposition step above 350 °C. The correspondence between this temperature and the T_max_ of cotton suggests that the FR efficacy of PAAs is related to their ability to intumesce following thermal decomposition and to create a physical barrier that blocks flame propagation. In order to investigate the intumescing ability of the PAAs considered, samples in the form of dry powders were heated to 350 °C in an oven in air and the residues analyzed by means of X-ray photoelectron spectroscopy (XPS). Figure 2 shows the morphology of the chars obtained by thermal treatment of M-GLU and M-LEU. The results obtained with all the other PAAs are shown in Figure S13. It is apparent that all samples significantly expanded under the action of oxygen and heat, forming the typical dark porous chars of intumescent materials, but with qualifications. PAAs bearing hydrophobic side chains, namely M-ALA, M-VAL and M-LEU, prevalently formed hollow structures delimited by thin and brittle crusts. All the other PAAs formed thick and soft muffin-shaped structures with almost no empty internal space. An XPS analysis demonstrated that all the PAA chars were C- and O-rich, as evidenced by the signals in the 284.0 and 285.4 eV range, corresponding to C-C and C=C signals, and those in the 286.6 and 287.5 eV range, corresponding to C-O and C=O signals [20]. Interestingly, all spectra showed a typical graphitic carbon signal at 284.5 eV [21], indicating that PAA intumescence generates rigid and stable aromatic structures. Furthermore, the XPS spectrum of M-GLU (Figure 2), as well as the spectrum of M-ASP and, to a lesser extent, the spectrum of M-GLN (Figure S13) showed an additional peak in the 288.0 and 290.0 eV range corresponding to the O-C=O signal, attributable to the formation of cyclic condensation products deriving from the esterification of the side COOH and CONH_2_ groups.

### 3.3. Ignitability of PAAs

**Figure 3:**
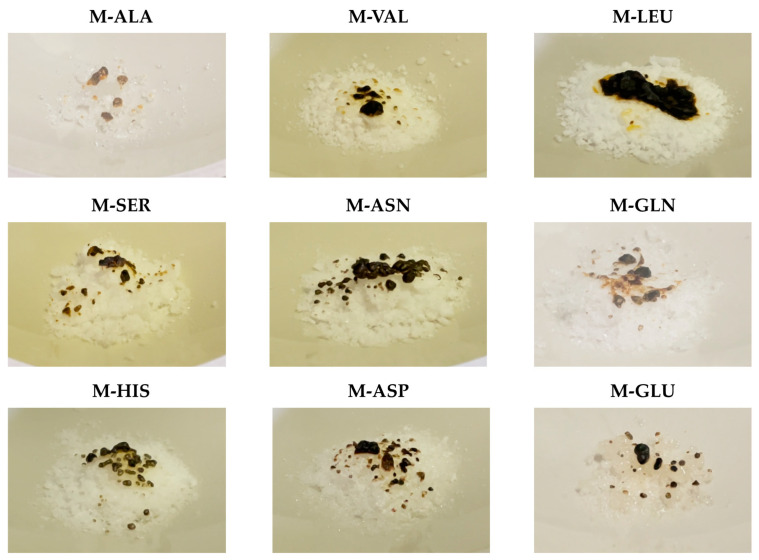
Snapshots of PAA ignition test residues.

The fire resistance of PAAs was evaluated by placing pulverized PAA samples in direct contact with a butane flame for 10 s. The results of the ignition tests are shown in Figure 3. The quantitative data on the RMF are shown in Table 4. As noted earlier with M-GLY [9], no PAA, apart from M-LEU, ignited after the flame was applied. They formed a black and porous surface crust, while the underlying powder remained unchanged and the overall RMF was above 98%. Instead, M-LEU underwent a flaming combustion for a duration of 25 s, leaving 90% RMF.

### 3.4. Morphological Characterization of Cotton Fabrics Treated with 7% Add-On PAAs

**Figure 4:**
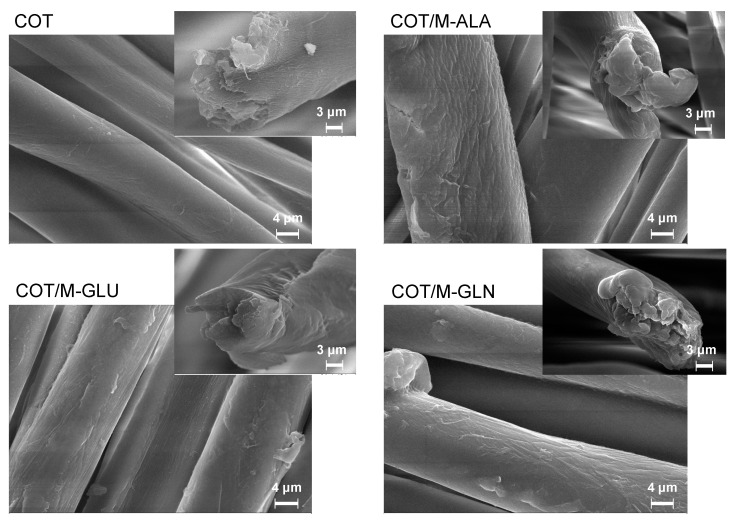
SEM micrographs (5000×) of untreated cotton (COT) and cotton fabrics treated with 7% add-on M-ALA, M-GLU and M-GLN. Insert magnification 10,000×.

Combustion tests were carried out on cotton fabrics impregnated with PAA aqueous solutions at pH 4.5 and then dried. The surface morphology of the cotton fabrics with 7% PAA add-on, whose structure was confirmed by ATR/FT-IR (Figure S12), was assessed by SEM observation. The SEM micrographs of untreated cotton and of cotton treated with three representative examples of PAAs, namely M-ALA, M-GLU and M-GLN, are shown in Figure 4. The morphologies of the cotton fabrics treated with all the remaining PAAs are shown in Figure S14. Both the untreated and the PAA-treated cotton fabrics had fibers that not only retained their individuality but had flat and smooth surfaces. In Figure 4, the inserts with 10,000× magnification, relative to the cut surface of a single fiber, show further interesting features. In the untreated cotton the single fibers had separated fibrils and an empty interstitial space, whereas, in the PAA-treated cotton samples, the inside of the fibers was completely filled and irregular protuberances protruding from the cut surfaces, demonstrating that during the impregnation phase the PAAs permeated the inside of the fibers and did more than just form a surface coating.

### 3.5. Thermal Characterization of PAA-Treated Cotton Fabrics

**Figure 5:**
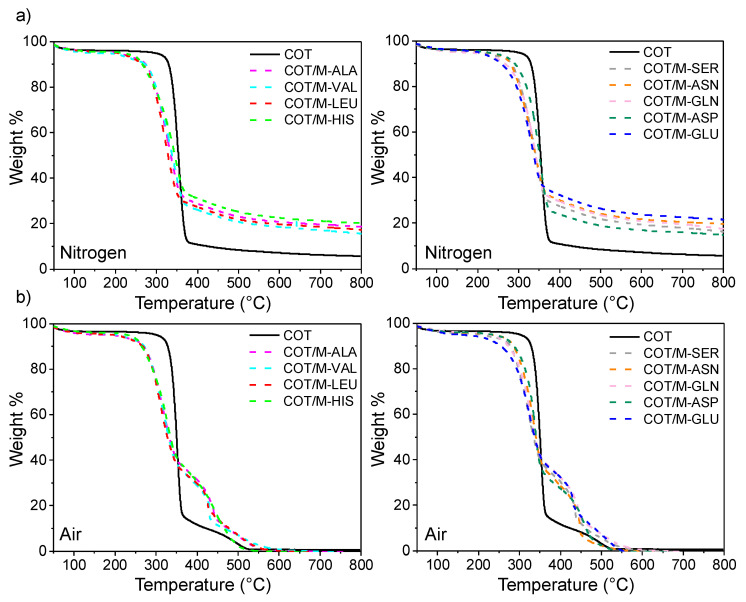
TG thermograms of untreated cotton and 7% add-on, PAA-treated cotton fabrics in nitrogen (**a**) and air (**b**).

PAA-treated cotton fabrics were thermally investigated using TGA in nitrogen and in the air (Figure 5a,b, respectively). The corresponding thermal data are shown in Table 5. Overall, all PAAs sensitized the thermal decomposition of the cotton, reducing T_onset10%_ in both atmospheres [22]. Above 360 °C in nitrogen and 350 °C in the air, all PAA-treated cotton samples were thermally more stable than the untreated cotton. In nitrogen, the RMF at 360 °C ranged from 30% to 40%, compared with a 10% value in untreated cotton at the same temperature, and then slowly decreased to 800 °C, at which temperature it ranged from 15% to 22%, a value three to four times higher than that in the untreated cotton. In the air, the thermal stability of the treated cotton samples was much higher than that of the untreated cotton, particularly in the range 350–450 °C, within which the PAAs underwent a significant intumescence. From 450 °C to 600 °C, the weight loss rate of PAA-treated cotton accelerated, although the RMF was generally higher than that of cotton. Not surprisingly, considering the low add-on values, the thermal stability of the different PAA-treated cotton specimens did not differ significantly from each other.

### 3.6. Combustion Studies of PAA-Treated Cotton Fabrics

As expected, based on the known behavior of M-GLY, none of the considered α-amino acid-derived PAAs were able to extinguish the flame in the vertical flame spread tests (VFSTs) to any add-on. Like M-GLY, they only reduced the rate of the fire spread and substantially increased the RMF at the end of the combustion tests. Therefore, in order to investigate the effect of the different α-amino acid residues on the efficacy of PAAs as FRs, PAA-treated cotton samples were subjected to horizontal flame spread tests (HFSTs) and to oxygen-consumption cone calorimetry tests to study the resistance to a radiant heat flux.

#### 3.6.1. Horizontal Flame Spread Tests (HFSTs)

**Figure 6:**
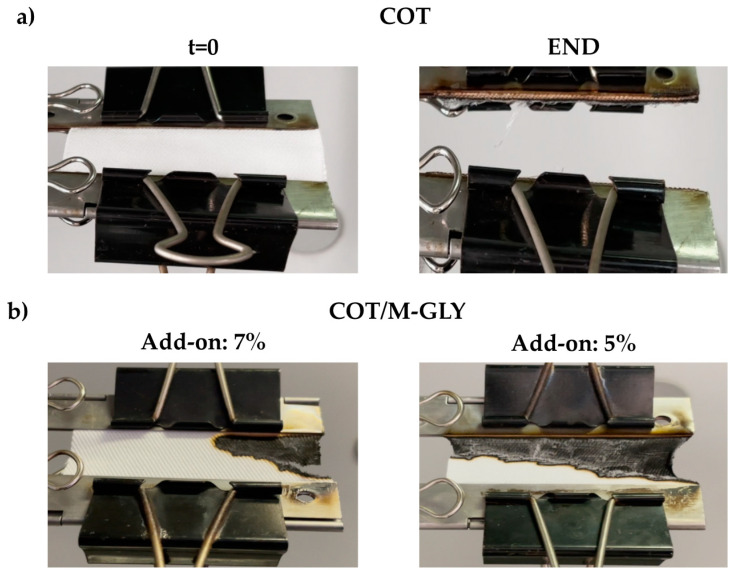
Snapshots of untreated cotton at the beginning and at the end of an HFST (**a**) and of M-GLY-treated cotton with add-ons, 7% and 5%, at the end of an HFST (**b**).

HFSTs were carried out using PAA-treated cotton specimens with both 7% and 5% add-ons. Their behavior was compared with the behavior of plain cotton that, once the flame was applied, burned rapidly, leaving no residue at the end of the test (Figure 6a). The flame resistance of the cotton treated with the M-GLY benchmark (Figure 6b) was also studied in the present work under exactly the same experimental conditions adopted for all the other PAAs.

**Figure 7:**
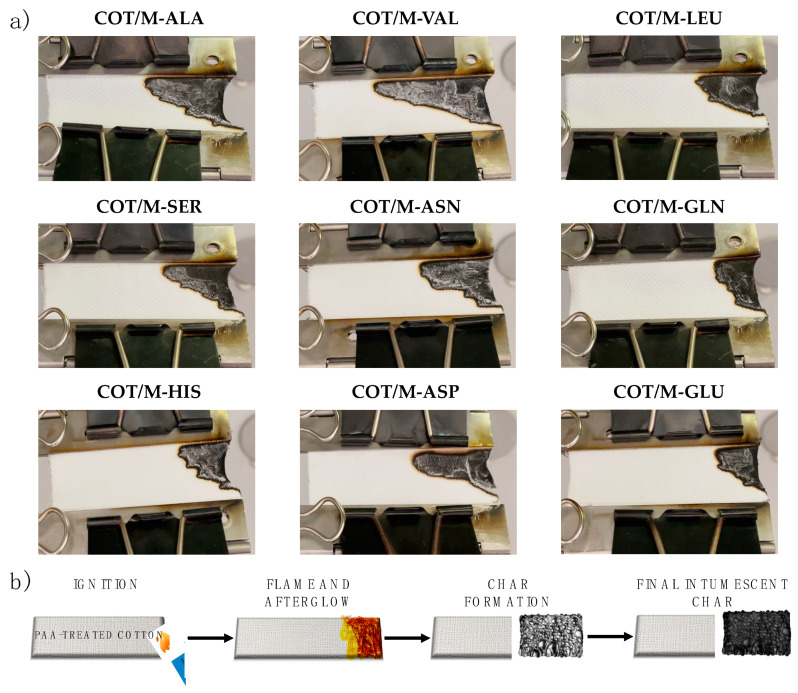
(**a**) Snapshots of the residues of 7% add-on, PAA-treated cotton samples after undergoing HFSTs. The PAA structures are reported in Table 2. (**b**) A sketch of the action mode of PAAs as intumescent FRs for cotton.

All cotton specimens with a 7% PAA add-on extinguished the flame (Figure 7a), although the flame spread with a slightly different burning time and the RMF ranged in a relatively wide range (64–90%, Table 6) within the series. By choosing the RMF value as a parameter, the efficacy of PAAs as FRs under these specific conditions was ranked in the following order: M-GLU > M-GLN > M-HIS > M-ASP, M-GLY ≈ M-LEU ≈ M-SER > M-ASN > M-ALA > M-VAL. It seems that the least effective PAAs, M-ALA and M-VAL, have hydrophobic alkyl lateral groups in the repeat unit, which are likely more susceptible to oxidation. An exception was provided by M-LEU that, based on the results of the ignition tests, was the most flammable of PAAs. M-HIS, M-ASP, M-GLY, M-LEU and M-SER exhibited high RMF values though they were lower than those of M-GLU and M-GLN, which proved to be the most effective PAAs in the ignition tests (Table 4). Interestingly, both PAAs were characterized by lateral groups with equal chain lengths but different functionalities. It can be observed that the highest performing PAAs, namely M-GLU, M-GLN, M-ASP and M-ASN, which do not have hydrocarbon side substituents, extinguished the flame in a shorter time than all the others, their combustion time ranging between 50 and 65 s, and, in the meantime, left higher RMF. In contrast, M-ALA and M-VAL, with hydrocarbon side substituents, left a lower RMF, 64 and 69%, combined with a longer combustion time.

A sketch of the action mode of PAAs as intumescent flame retardants for cotton is shown in Figure 7b.

**Figure 8:**
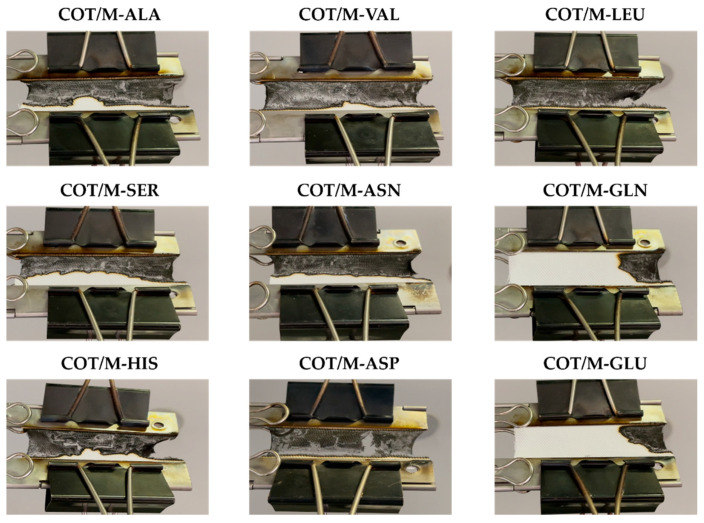
Snapshots of the residues of 5% add-on, PAA-treated cotton samples after undergoing HFSTs. The PAA structures are reported in Table 2.

Among 5% add-on, PAA-treated cotton samples, only COT/M-GLU and COT/M-GLN were able to extinguish the flame (Figure 8). All other samples burnt leaving a lower RMF and exhibiting a higher combustion time. They also showed the highest RMF values (84% and 74%, respectively) and their combustion times did not differ significantly from those of the same samples with 7% add-on (Table 7). Even if they burnt completely, all other samples showed much higher combustion times and RMF values than plain cotton (Table 7). Among them, the most performant were COT/M-ASN and COT/SER with 32% and 25% RMF, respectively, the latter exhibiting the lowest flame spread rate. All the other samples showed a similar performance.

**Figure 9:**
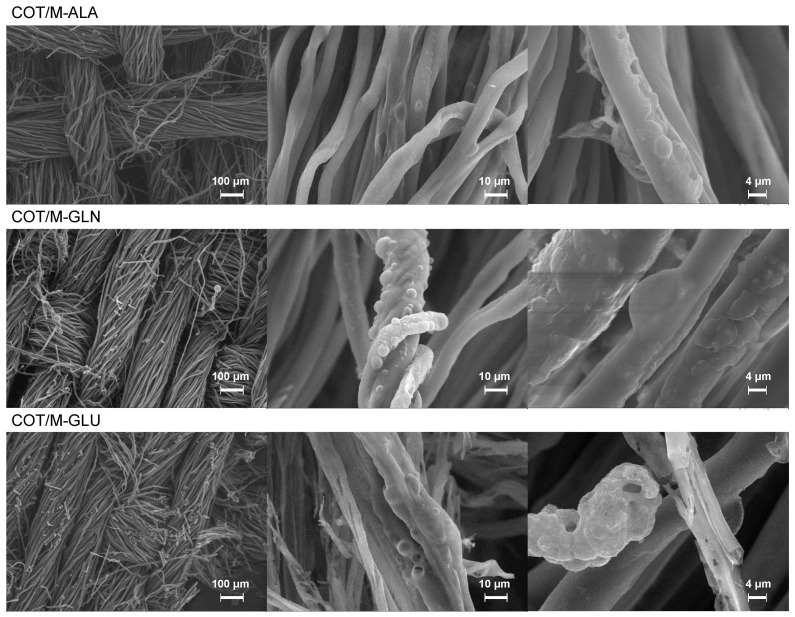
SEM micrographs of 7% add-on COT/MBA-ALA, COT/MBA-GLN and COT/MBA-GLU residues from HFSTs.

The morphology of the residues left by the PAA-cotton fabrics, with 7% add-on after undergoing HFSTs, was observed by SEM. Figure 9 shows representative examples of the results obtained, specifically those of COT/M-ALA, COT/M-GLN and COT/M-GLU. The morphologies of the residues of the other COT/PAA samples are shown in Figures S15 and S16. It is apparent that the burnt areas of the self-extinguished fabrics maintained the original shape and texture of cotton and the fibers appeared to be intact, notwithstanding combustion. At higher magnifications, numerous microbubble-rich zones were visible in the internal fibrils, demonstrating that the intumescence of PAA-treated cotton is a phenomenon that spread evenly among the fibers.

#### 3.6.2. Cone Calorimetry Tests

**Figure 10:**
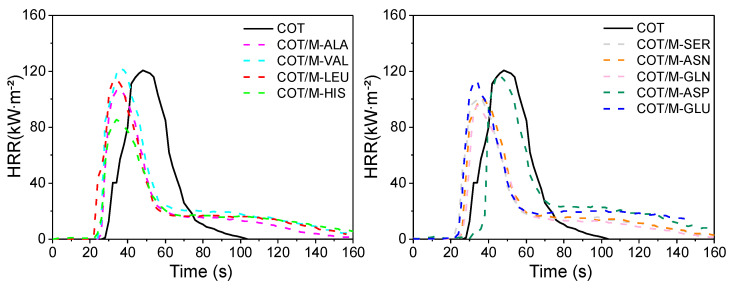
Heat release rate curves of untreated and 7% add-on, PAA-treated cotton fabrics.

Oxygen-consumption cone calorimetry tests are best suited to simulate a realistic fire scenario [19]. In these tests, PAA-treated cotton fabrics were exposed to a 35 kW m^−2^ heat flux, similar to what is normally found in developing fires [23]. Figure 10 compiles the heat release rate (HRR) curves of control and treated fabrics. Observing the HRR curves of PAA-treated cotton samples, it is noted that after about 60 s these curves differed from that of untreated cotton and were typical of a thick charring material [23].

Combustion parameters such as the time to ignition (TTI), peak of heat release (pKHRR), total heat release (THR), and residual mass fraction (RMF) are reported in Table 8. Due to the low PAA add-ons used (7%), the TTI, FPI and THR values, as well as the CO and CO_2_ release reduction (data not shown) do not differ significantly from those of cotton. The TTI values of all the PAA-treated cotton samples shown are lower than the TTI of untreated cotton. Apparently, the presence of the PAA coatings, even at low add-on (4%), sensitizes cotton towards thermal decomposition. This is not at odds with the overall FR properties of PAAs, as their role is to modify the thermo-oxidative mechanism of cotton by creating a protective char layer. This is in line with the results of the flammability tests of the PAAs themselves, which did not ignite by direct impingement from a propane flame, apart from *L*-Leucine (see above), but were soon protected by a layer of char on the surface.

However, in general, all PAAs were effective in protecting cotton fabrics from combustion, as evidenced by the significant RMF values (2.5–5.5% compared with 0% for cotton) and reduction in the pKHRR values (−7–−33%) (Table 8). The RMF values obtained in the cone calorimetry measurements varied between 2.5% and 5.5%, whereas in TGA analyses no residues were left at 800 °C. It should be observed, however, that the cone calorimetry and TGA experiments were carried out with a different configuration and under remarkably different experimental conditions. In particular, the heating rate used in the cone calorimetry experiments was higher by one order of magnitude, compared to the TGA experiments. Therefore, the kinetics of char formation, as well as the morphology, and mechanical and thermal stability of the char, are expected to be different.

## 4. Conclusions 

The aim of this work was to ascertain whether the ability of intumescing under the effect of oxygen and heating, and to act as efficient flame retardant (particularly in HFST, previously observed for M-GLY, derived from the polyaddition of MBA with glycine), were general features of the PAAs derived from α-amino acids or were limited to a few of them. The purpose was to establish the correlations between the structural characteristics of PAAs, in particular the nature of the amino acid residues, on their resistance to combustion. To accomplish this goal, nine PAAs were synthesized from the polyaddition of MBA with different α-amino acids: *L*-alanine, *L*-valine, *L*-leucine, *L*-histidine, *L*-serine, *L*-asparagine, *L*-glutamine, *L*-aspartic acid, and *L*-glutamic acid. Their behavior was compared with that of M-GLY, chosen as a benchmark.

Like M-GLY, all PAAs were found to be non-flammable when subjected to ignition tests that applied a butane flame. Furthermore, their TG curves in air were very similar to those in nitrogen up to 350 °C. Above this temperature, the oxidation of the residues of the decomposition products, formed in the previous phase in air, took place.

HFSTs and VFSTs were performed on cotton strips impregnated with PAA solutions and then dried. The morphology of the PAA-treated fabrics, analyzed by SEM, showed a regular distribution of the PAA coating over the entire fabric up to the inside of the fibers. All the tested PAAs behaved similarly to M-GLY in other respects both in HFSTs and VFSTs. None of the PAAs extinguished the flame in VFSTs even at high add-ons, but all of them left a substantial residue. However, all of the PAAs efficiently extinguished the flame in HFSTs at an add-on above 7%; the same applied to M-GLU and M-GLN at a 5% add-on. By choosing RMF as a parameter to classify PAAs as FR, it turned out that, even with a 7% add-on, M-GLU and M-GLN were the best performing PAAs, whereas M-ALA and M-VAL were the least effective. The burnt areas of all the samples, analyzed by SEM, maintained the original shape and texture of cotton and the fibers appeared intact. At higher magnifications, numerous microbubble-rich zones were visible in the internal fibrils, demonstrating that the intumescence of PAA-treated cotton is a phenomenon spread evenly among the cotton fibers.

The results of the cone calorimetry tests carried out on the cotton fabrics with a 7% PAA add-on showed that, at this low PAA amount, the TTI, FPI and THR values did not differ significantly from those of cotton, but that all PAAs were effective in protecting cotton fabrics from combustion, as evidenced by the significant RMF values and the reduction in the pKHRR values. 

The analysis of the chars obtained after heating in the air at 350 °C provided useful information for interpreting the role of the PAAs’ repeat unit structure in determining their behavior during the combustion process of cotton. All samples significantly intumesced upon heating in air, forming dark porous chars. However, M-ALA, M-VAL and M-LEU, which had easily oxidized alkyl groups in the repeat units, formed prevalently hollow structures delimited by thin brittle crusts. All the other PAAs, particularly M-GLU and M-GLN, formed thick and soft muffin-shaped structures with almost no empty internal space. An XPS analysis demonstrated that all the PAA chars were C- and O-rich and showed a graphitic carbon signal, indicating that PAA intumescence generated rigid and thermally stable aromatic structures. The superior FR performance of M-GLU and M-GLN, carrying lateral groups with same number of methylene groups, was expressly attributed to their ability to form cyclic intermediates capable of forming stable graphitic char. 

It may be reasonably concluded that α-amino acid-derived PAAs are a sub-class of polymers with remarkable potential as intumescent FRs for cotton. Interestingly, the phenomenon of the intumescence of PAAs is particularly significant at 350 °C, which corresponds precisely to the T_max_ of cotton. In these conditions, the porous crust formed by heating the PAA coating under the effect of oxygen exerts its protective action on cotton by insulating it and modifying its decomposition pattern. The intimate contact of the PAA coating on the inside of cotton fibers can contribute to their performance.

## Figures and Tables

**Table 1:** Amounts of reagents used in the synthesis of PAAs ^1^.

PAA ^2^	[α-Amino Acid] ^3^ (g)	LiOH∙H_2_O (g, mol)	H_2_O (mL)
M-GLY	7.5	4.2, 0.1	35
M-ALA	8.9	4.2, 0.1	40
M-VAL	11.7	4.2, 0.1	42
M-LEU	13.1	4.2, 0.1	45
M-HIS	15.5	4.2, 0.1	53
M-SER	10.5	4.2, 0.1	45
M-ASN	13.2	4.2, 0.1	50
M-GLN	14.6	4.2, 0.1	60
M-ASP	13.3	8.4, 0.2	55
M-GLU	14.6	8.4, 0.2	60

^1^ MBA amount: 15.4 g (0.1 mol). ^2^ M stands for MBA and the following three letters for the a-amino acid code, as in Scheme 1. ^3^ Amount of the a-amino acid: 0.1 mol.

**Table 2:** Structure of the α-amino acid-derived PAA repeat units.

PAA	Structure of the Repeat Unit	PAA	Structure of the Repeat Unit
M-GLY	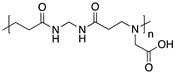	M-ALA	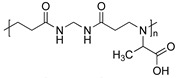
M-VAL	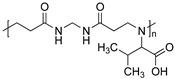	M-LEU	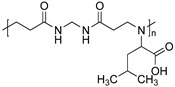
M-HIS	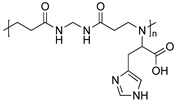	M-SER	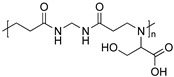
M-ASN	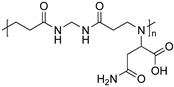	M-GLN	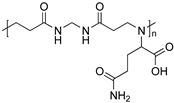
M-ASP	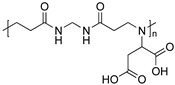	M-GLU	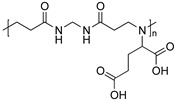

**Table 3:** Thermal data of α-amino acid-derived PAAs in nitrogen and air by thermogravimetric analysis.

Sample	T_onset10%_^1^ (°C)	T_max1_^2^ (°C)	T_max2_^3^ (°C)	RMF_800_^4^ (%)
Nitrogen				
M-GLY	152	270	-	12
M-ALA	160	302	-	16
M-VAL	223	275	-	14
M-LEU	116	270	-	9
M-HIS	135	260	-	20
M-SER	129	245	-	18
M-ASN	126	308	-	26
M-GLN	145	315	-	21
M-ASP	157	230	-	26
M-GLU	172	340	-	26
Air				
M-GLY	205	260	610	3
M-ALA	150	290	550	7
M-VAL	229	274	540	9
M-LEU	102	256	510	6
M-HIS	123	255	560	1
M-SER	126	236	541	3
M-ASN	149	254	560	5
M-GLN	159	270	500	1
M-ASP	155	225	616	4
M-GLU	177	320	530	4

^1^ Onset decomposition temperature at 10% weight loss. ^2^ First temperature at maximum weight loss rate. ^3^ Second temperature at maximum weight loss rate. ^4^ Residual mass fraction at 800 °C.

**Table 4:** PAA ignition test data.

Sample	RMF (%) ^1^	Sample	RMF (%) ^1^
M-GLY	98.0	M-SER	98.9
M-ALA	98.6	M-ASN	98.9
M-VAL	99.5	M-GLN	99.5
M-LEU	90.0	M-ASP	98.6
M-HIS	98.8	M-GLU	99.7

^1^ RMF: residual mass fraction.

**Table 5:** Thermal data of untreated cotton and 7% add-on, PAA-treated cotton fabrics in nitrogen and air obtained by thermogravimetric analysis.

Sample	T_onset10%_^1^ (°C)	T_max1_^2^ (°C)	T_max2_^3^ (°C)	RMF_800_^4^ (%)
Nitrogen				
COT	329	355	-	5.5
COT/M-ALA	274	332	-	19.0
COT/M-VAL	265	340	-	15.5
COT/M-LEU	262	321	-	17.0
COT/M-HIS	264	347	-	20.0
COT/M-SER	277	343	-	16.0
COT/M-ASN	278	342	-	20.0
COT/M-GLN	266	341	-	17.5
COT/M-ASP	290	354	-	15.0
COT/M-GLU	256	333	-	21.5
Air				
COT	329	352	480	-
COT/M-ALA	270	315	440	-
COT/M-VAL	272	324	429	-
COT/M-LEU	271	309	426	-
COT/M-HIS	265	327	441	-
COT/M-SER	276	321	435	-
COT/M-ASN	287	336	445	-
COT/M-GLN	273	327	442	-
COT/M-ASP	291	340	457	-
COT/M-GLU	256	323	431	-

^1^ Onset decomposition temperature at 10% weight loss. ^2^ First temperature at maximum weight loss rate. ^3^ Second temperature at maximum weight loss rate. ^4^ Residual mass fraction at 800 °C.

**Table 6:** Combustion data of untreated and PAA-treated cotton fabrics with 7% add-on obtained from HFSTs.

Sample	Combustion Time ^1^ (s)	Extinguishment	RMF ^2^ (%)
COT	53	NO	<1
COT/M-GLY	80	YES	81
COT/M-ALA	94	YES	69
COT/M-VAL	70	YES	64
COT/M-LEU	82	YES	80
COT/M-SER	65	YES	79
COT/M-ASN	80	YES	77
COT/M-GLN	51	YES	87
COT/M-HIS	65	YES	84
COT/M-ASP	50	YES	81
COT/M-GLU	54	YES	90

^1^ Combustion time ± 1 s. ^2^ RMF ± 1.0%.

**Table 7:** Combustion data of untreated and PAA-treated cotton fabrics with 5% add-on obtained from HFSTs.

Sample	Combustion Time ^1^ (s)	Extinguishment	RMF ^2^ (%)
COT	53	NO	<1
COT/M-GLY	120	NO	25
COT/M-ALA	93	NO	23
COT/M-VAL	92	NO	18
COT/M-LEU	111	NO	17
COT/M-SER	102	NO	25
COT/M-ASN	81	NO	32
COT/M-GLN	66	YES	74
COT/M-HIS	96	NO	17
COT/M-ASP	84	NO	17
COT/M-GLU	60	YES	84

^1^ Combustion time ± 1 s. ^2^ RMF ± 1.0%.

**Table 8:** Combustion data of untreated and PAA-treated cotton fabrics (add-on: 7%) from cone calorimetry.

Sample	TTI (s) ^1^	pKHRR ^2^ (kW m^−2^) (Reduction %)	FPI^3^ (sm^2^kW^−1^)	THR ^4^ (MJ m^−2^)	RMF ^5^ (%)
COT	28 ± 1	131 ± 5	0.21 ± 0.01	3.5 ± 0.2	0
COT/M-ALA	25 ± 1	114 ± 5 (−13)	0.22 ± 0.01	3.3 ± 0.2	4.0 ± 0.2
COT/M-VAL	25 ± 1	122 ± 0 (−7)	0.20 ± 0.01	4.1 ± 0.2	2.5 ± 0.7
COT/M-LEU	24 ± 3	121 ± 3 (−8)	0.20 ± 0.01	3.8 ± 0.2	4.0 ± 0.4
COT/M-SER	23 ± 4	110 ± 9 (−16)	0.21 ± 0.01	3.4 ± 0.2	4.0 ± 0.4
COT/M-ASN	24 ± 3	101 ± 7 (−23)	0.24 ± 0.01	3.4 ± 0.2	4.5 ± 0.7
COT/M-GLN	25 ± 1	96 ± 1 (−27)	0.26 ± 0.01	2.9 ± 0.1	5.5 ± 0.7
COT/M-HIS	23 ± 1	88 ± 13 (−33)	0.26 ± 0.01	3.3 ± 0.2	3.5 ± 0.7
COT/M-ASP	26 ± 1	117 ± 6 (−10)	0.22 ± 0.01	4.2 ± 0.2	3.0 ± 0.4
COT/M-GLU	25 ± 1	119 ± 22 (−9)	0.21 ± 0.01	4.0 ± 0.2	3.5 ± 0.7

^1^ Time to ignition. ^2^ Peak of heat release rate. ^3^ Fire performance index. ^4^ Total heat release. ^5^ Residual mass fraction.

## Supplementary Materials

The following are available online at https://www.mdpi.com/article/10.3390/polym13213714/s1, Figures S1–S10: ^1^H-NMR spectra of PAAs with assignments. Figures S11 and S12: FT-IR/ATR spectra of PAAs and PAA-treated cotton fabrics. Figure S13: XPS spectra of PAAs. Figure S14: SEM micrographs of PAA-treated cotton fabrics (add-on: 7%). Figures S15 and S16: SEM micrographs of COT/PAA (add-on: 7%) residues deriving from horizontal flame spread tests.
