# Supplementary material for: Polyamidoamines Derived from Natural α-Amino Acids as Effective Flame Retardants for Cotton
Source: Polymers (Basel). 2021 Oct 28;13(21):3714. doi: 10.3390/polym13213714 (PMC8588117; doi:10.3390/polym13213714)
Supplement: Supplementary file 1 [file polymers-13-03714-s001.zip › polymers-1432827-supplementary.pdf]

Supplementary Materials

Polyamidoamines derived from natural α-amino acids as effective flame retardants for cotton

Alessandro Beduini,[1] Federico Carosio,[2] Paolo Ferruti,[1] Elisabetta Ranucci,[1] Jenny Alongi[1*]

[1] Dipartimento di Chimica, Università degli Studi di Milano, via C. Golgi 19, 20133 Milano, Italy alessandro.beduini@unimi.it (A.B.); paolo.ferruti@unimi.it (P.F); elisabetta.ranucci@unimi.it (E.R.)

[2] Dipartimento di Scienza Applicata e Tecnologia, Politecnico di Torino, Alessandria campus, viale T. Michel, 15121 Alessandria, Italy; federico.carosio@polito.it (F.C.)

* Correspondence: jenny.alongi@unimi.it (J.A.); Tel.: +39–02-50314108

Pages S1-S11

Figures S1-S10: ^{1}H-NMR spectra of PAAs with assignments.

Figures S11-S12: FT-IR/ATR spectra of PAAs and PAA-treated cotton fabrics.

Figure S13: XPS spectra of PAAs.

Figure S14: SEM micrographs of PAA-treated cotton fabrics (add-on: 7%).

Figures S15 and S16: SEM micrographs of COT/PAA (add-on: 7%) residues deriving from horizontal flame spread tests.

^{1}H-NMR characterization

All PAAs were characterized by 1H-NMR spectroscopy, using a Brüker Avance DPX-400 NMR spectrometer (Milano, Italy) operating at 400.13 MHz. Number of scans 32, relaxation delay, d1, 10.0 s, receiver gain automatically measured and set by the instrument. Analyses were conducted in D_2O, adjusting the pH with D_2O solutions of DCl to pH 4.0.

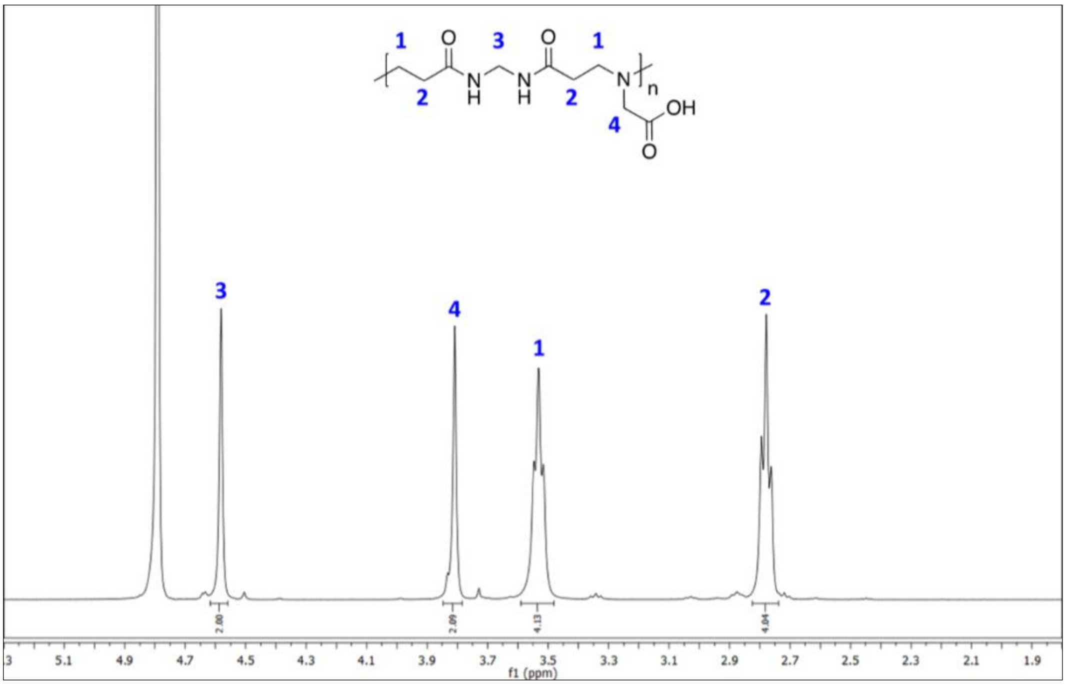

Figure S1. ^{1}H-NMR of M-GLY at pH 4.0.

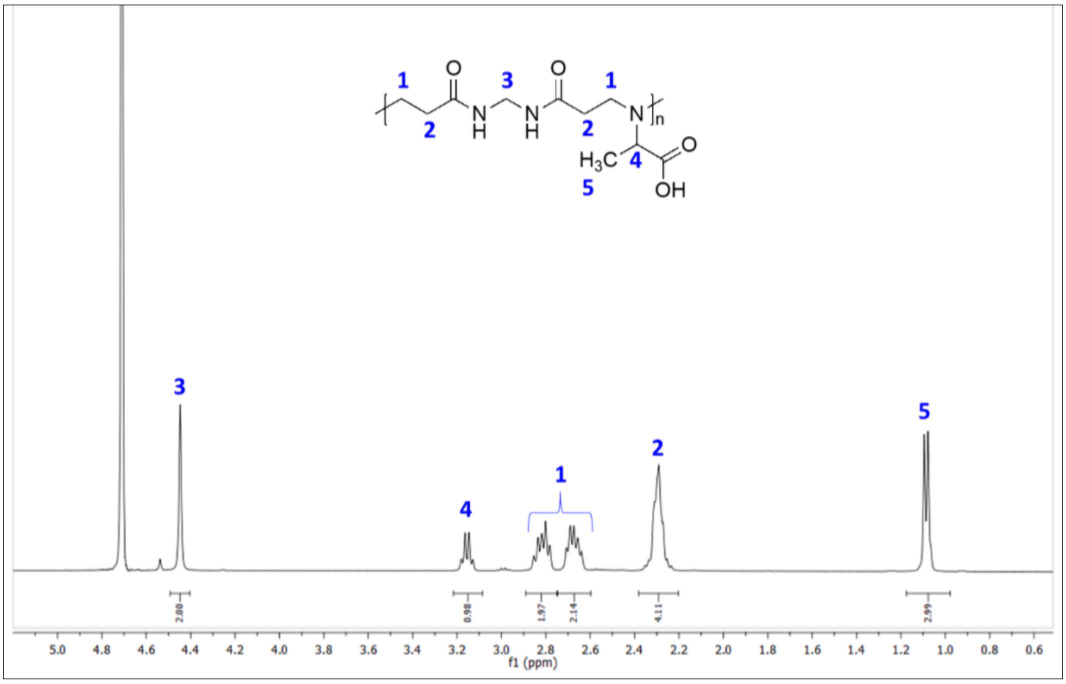

Figure S2. ^{1}H-NMR of M-ALA at pH 4.0.

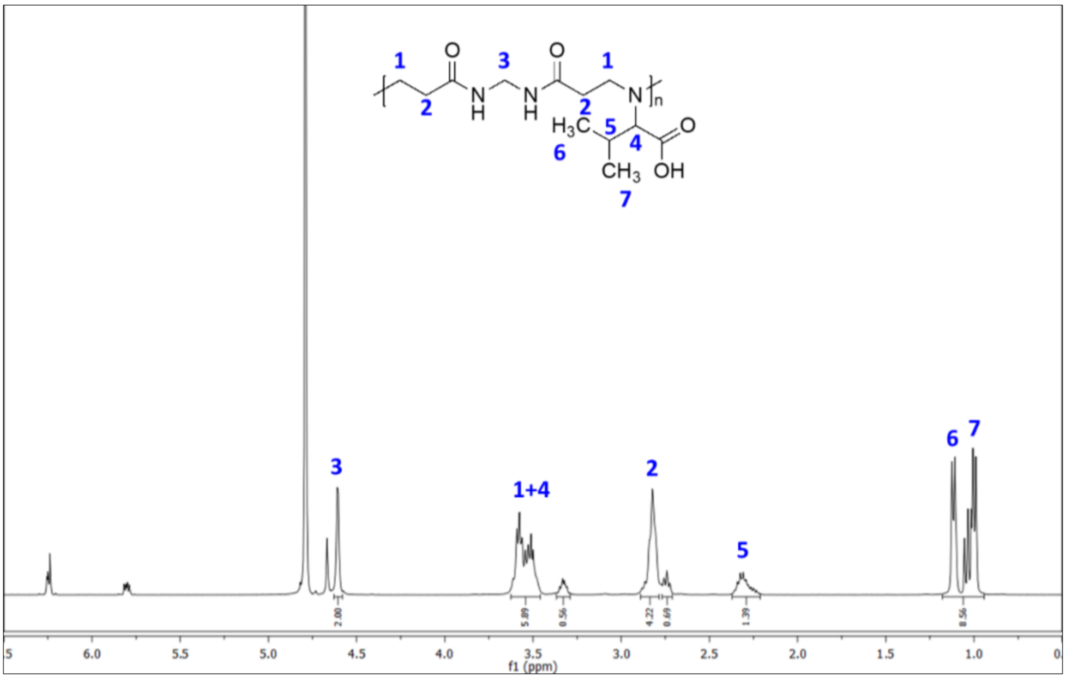

Figure S3. ^{1}H-NMR of M-VAL at pH 4.0.

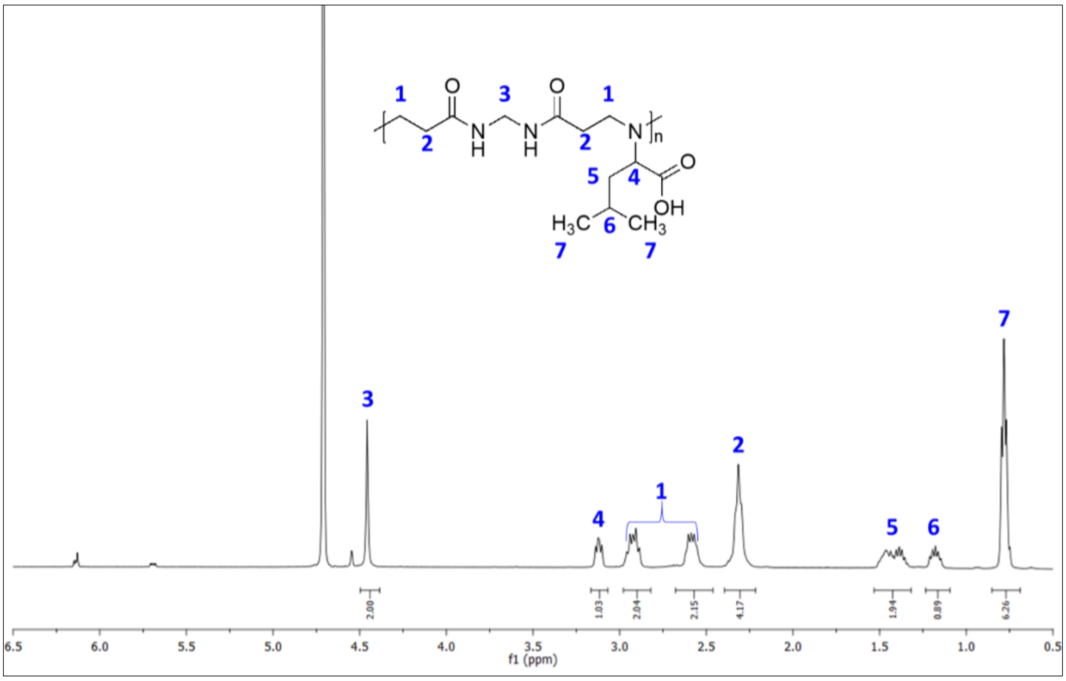

Figure S4. ^{1}H-NMR of M-LEU at pH 4.0.

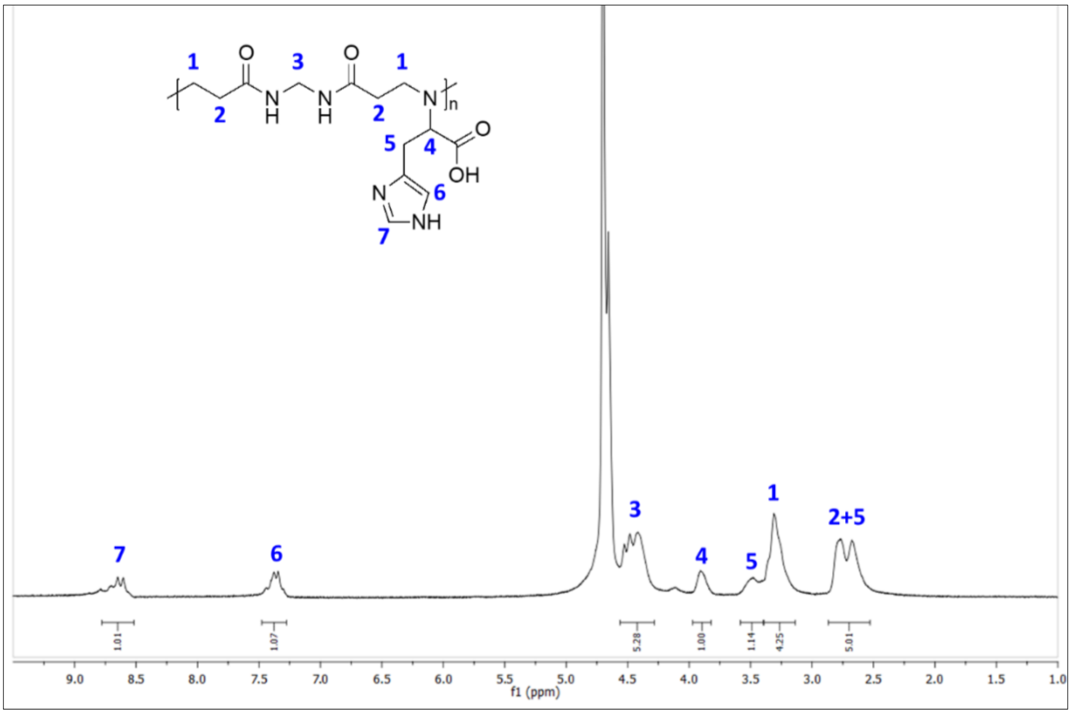

Figure S5. ^{1}H-NMR of M-HIS at pH 4.0.

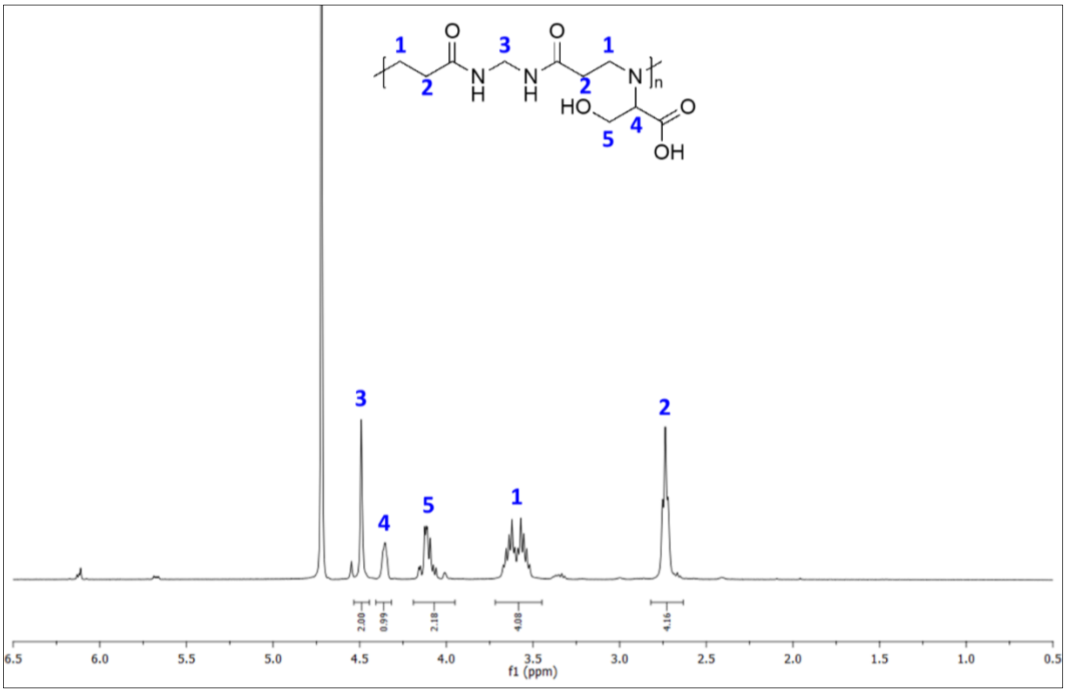

Figure S6. ^{1}H-NMR of M-SER at pH 4.0.

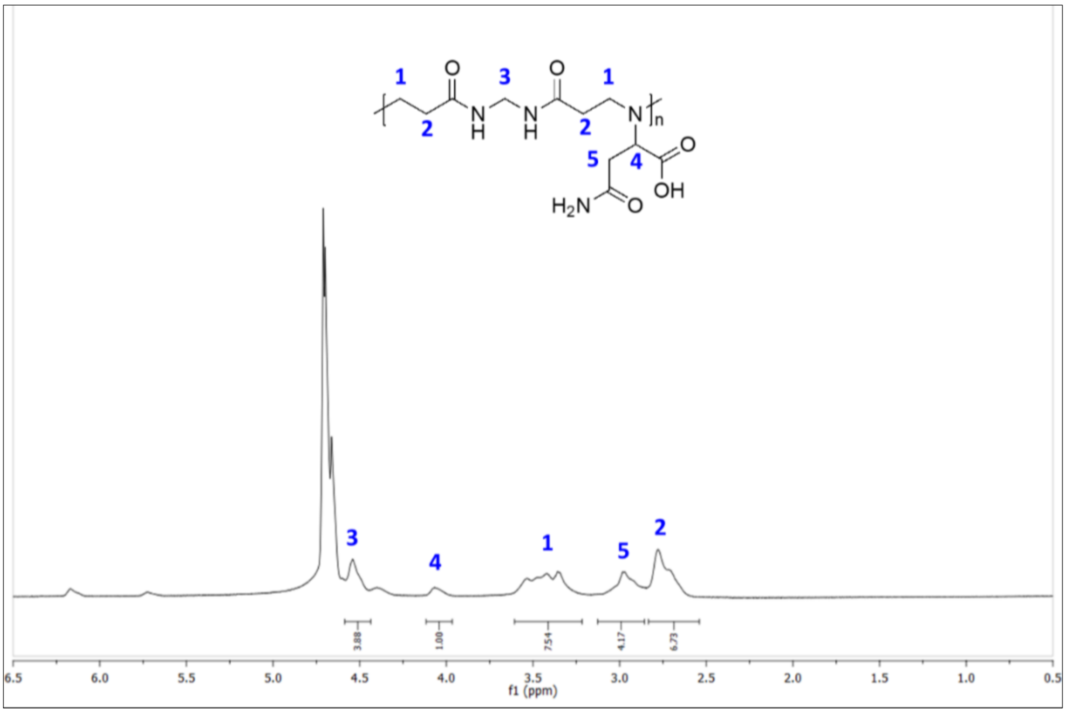

Figure S7. ¹H-NMR of M-ASN at pH 4.0.

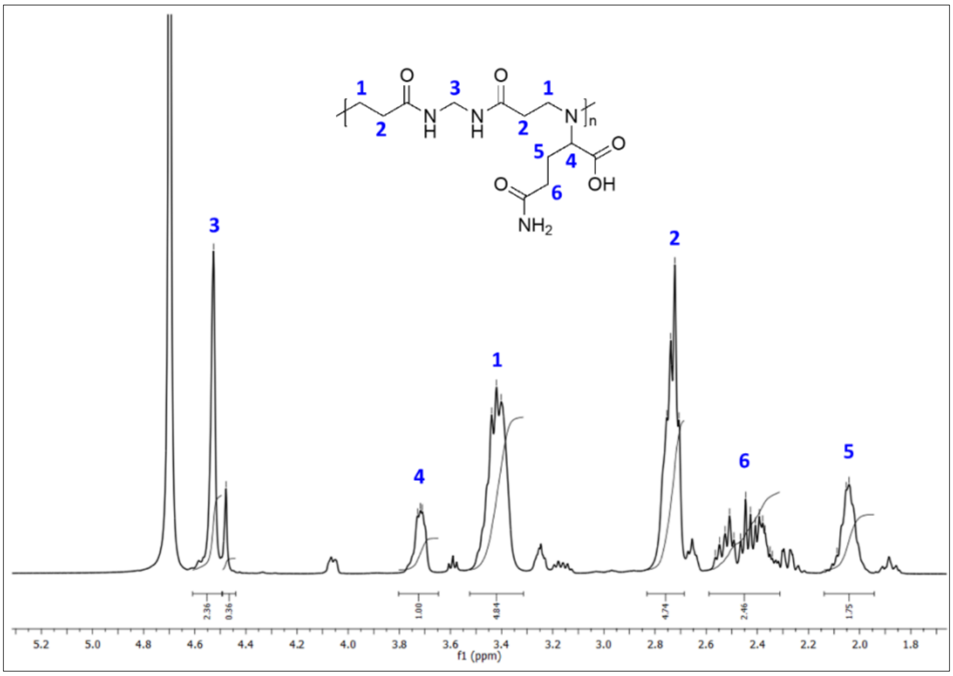

Figure S8. ¹H-NMR of M-GLN at pH 4.0.

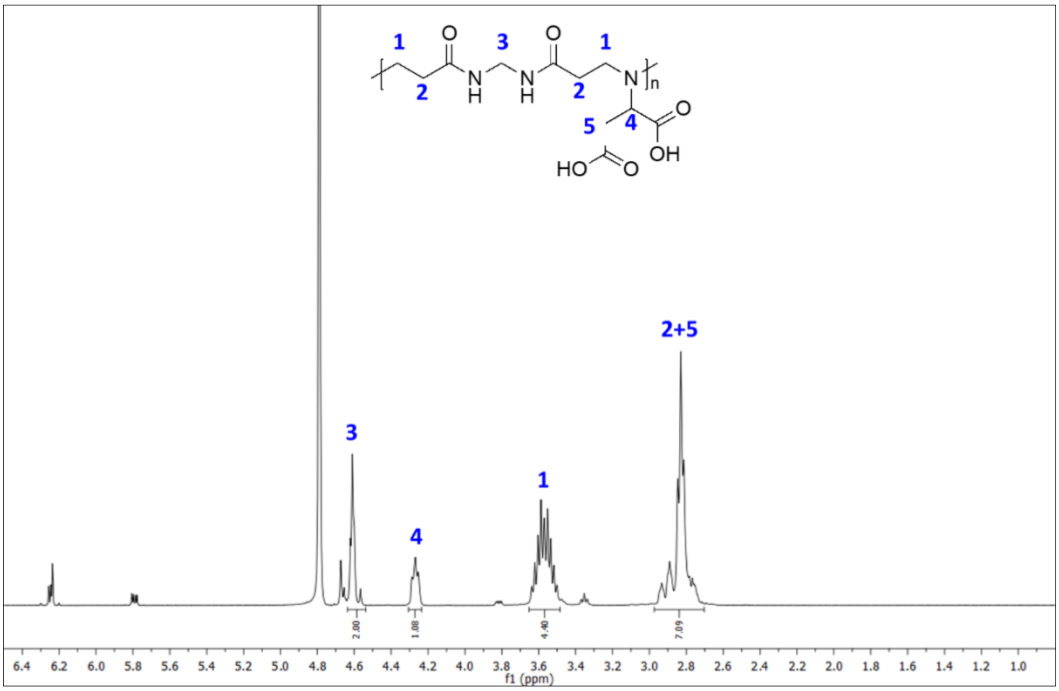

Figure S9. ^{1}H-NMR of M-ASP at pH 4.0.

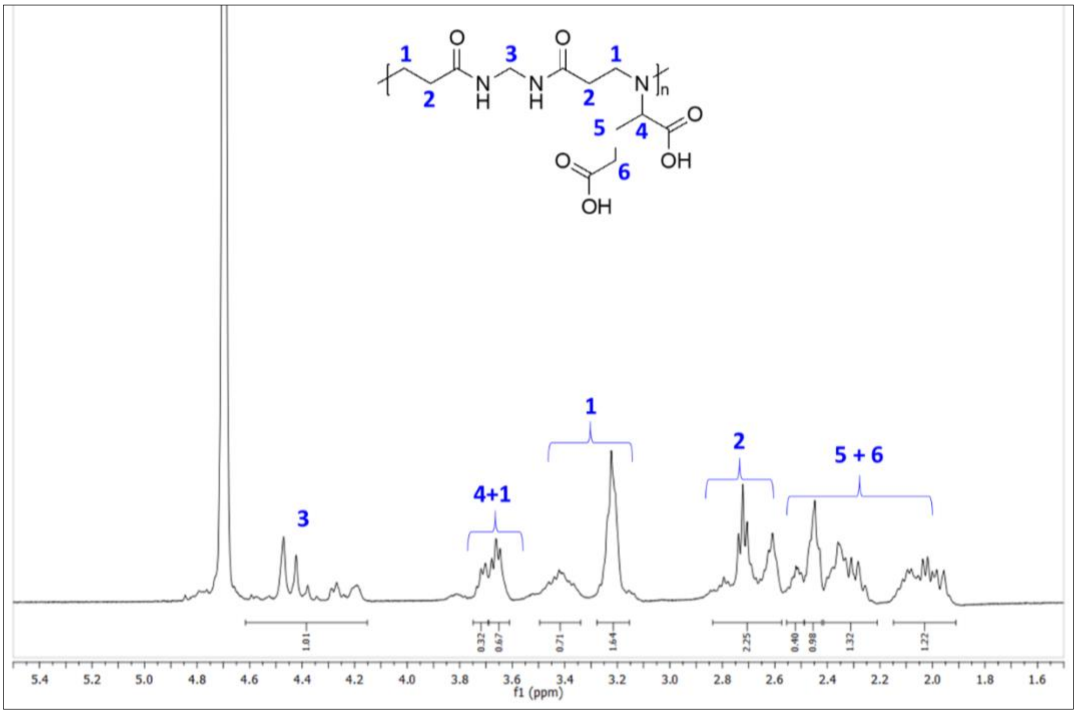

Figure S10. ^{1}H-NMR of M-GLU at pH 4.0.

FT-IR/ATR characterization

All PAAs were analyzed by attenuated total reflectance (ATR) Fourier transform infrared spectroscopy (FT-IR). FT-IR/ATR spectra were recorded at room temperature, in the 4000 - 380 cm^{-1} wavenumber range, with 32 scans and 4 cm^{-1} resolution, using a Perkin-Elmer Frontier FT-IR/FIR spectrophotometer (Milano, Italy), equipped with a diamond crystal characterized by a penetration depth of 1.66 μm.

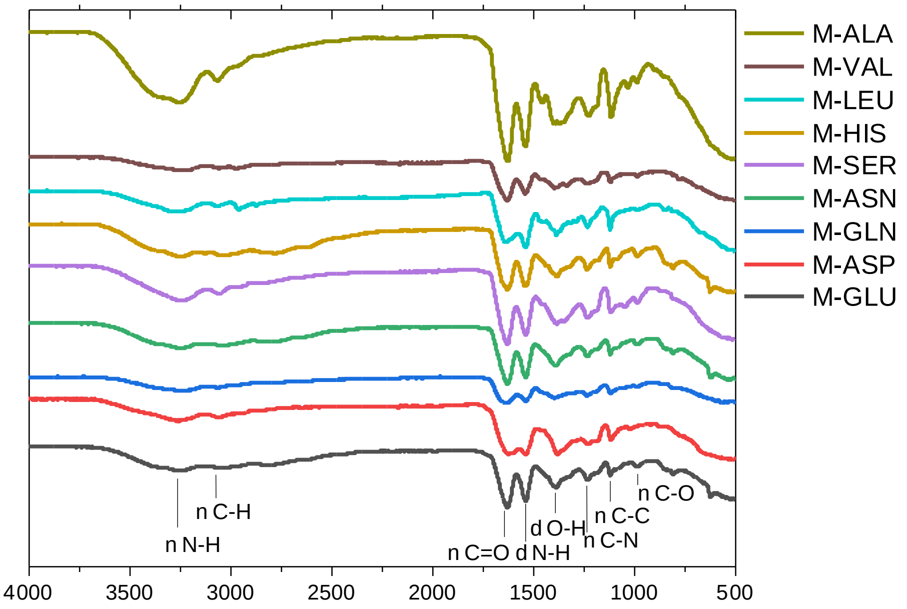

Figure S11. FT-IR/ATR spectra of PAAs.

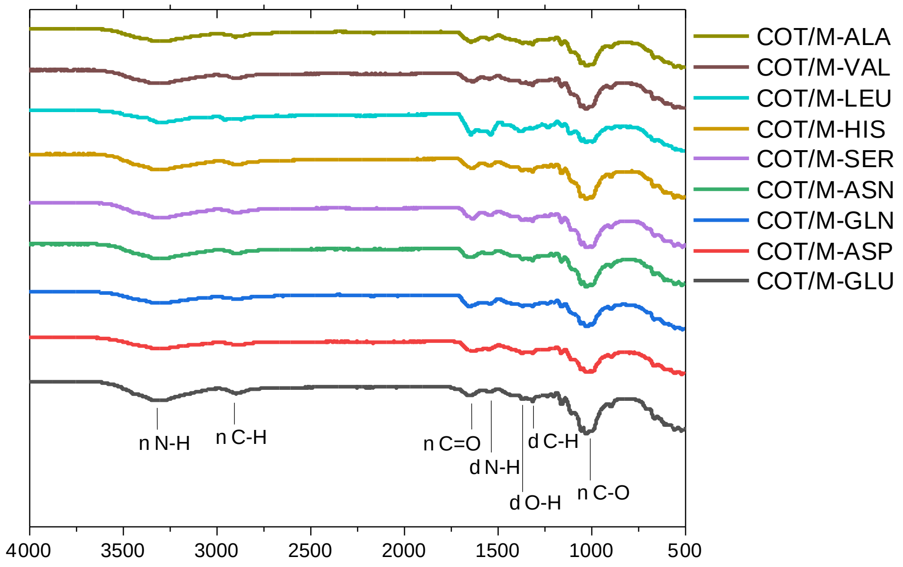

Figure S12. FT-IR/ATR spectra of PAA-treated cotton fabrics.

XPS analysis

PAA residues heated at 350 °C in an oven were analyzed using an X-ray photoelectron spectrometer (XPS) equipped with an Al K radiation monochromatic source (1486.6 eV) and manufactured by Surface Science Instruments – Biolin Scientific UK (Manchester, UK).

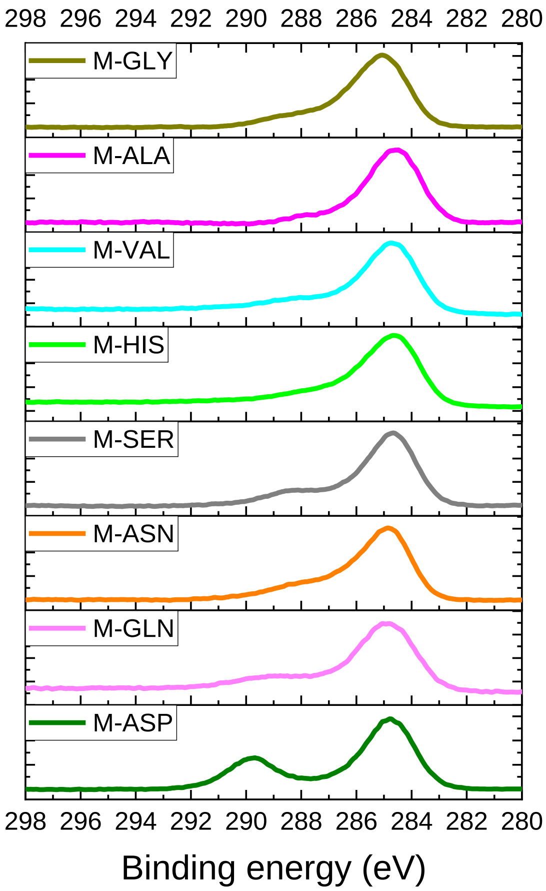

Figure S13. XPS spectra of PAAs.

Morphological characterization of PAA-treated cotton fabrics by SEM

The surface morphology of untreated and treated cotton fabrics was analyzed by a EVO 15 equipped with a ULTIM MAX 40 probe scanning electron microscope (SEM) manufactured by Zeiss (Ramsey, NJ, USA) and operating at 8.5 mm working distance, under 5 kV beam voltage. A fabric piece (5 × 5 mm^2) was fixed to conductive carbon adhesive tape and then gold-metallized.

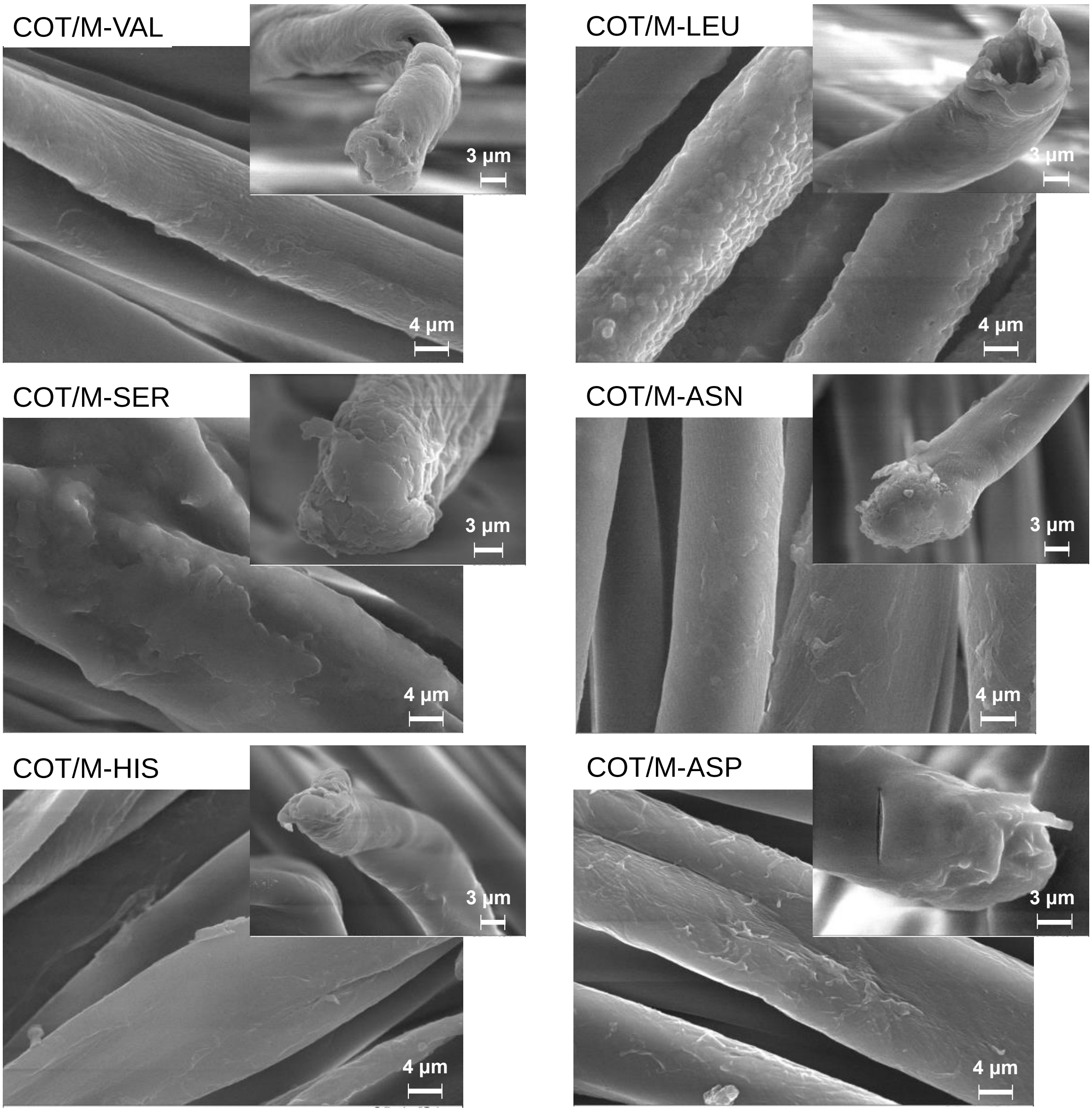

Figure S14. SEM micrographs of COT/M-VAL, COT/M-LEU, COT/M-SER, COT/M-ASN, COT/M-HIS and COT/M-ASP (add-on: 7%).

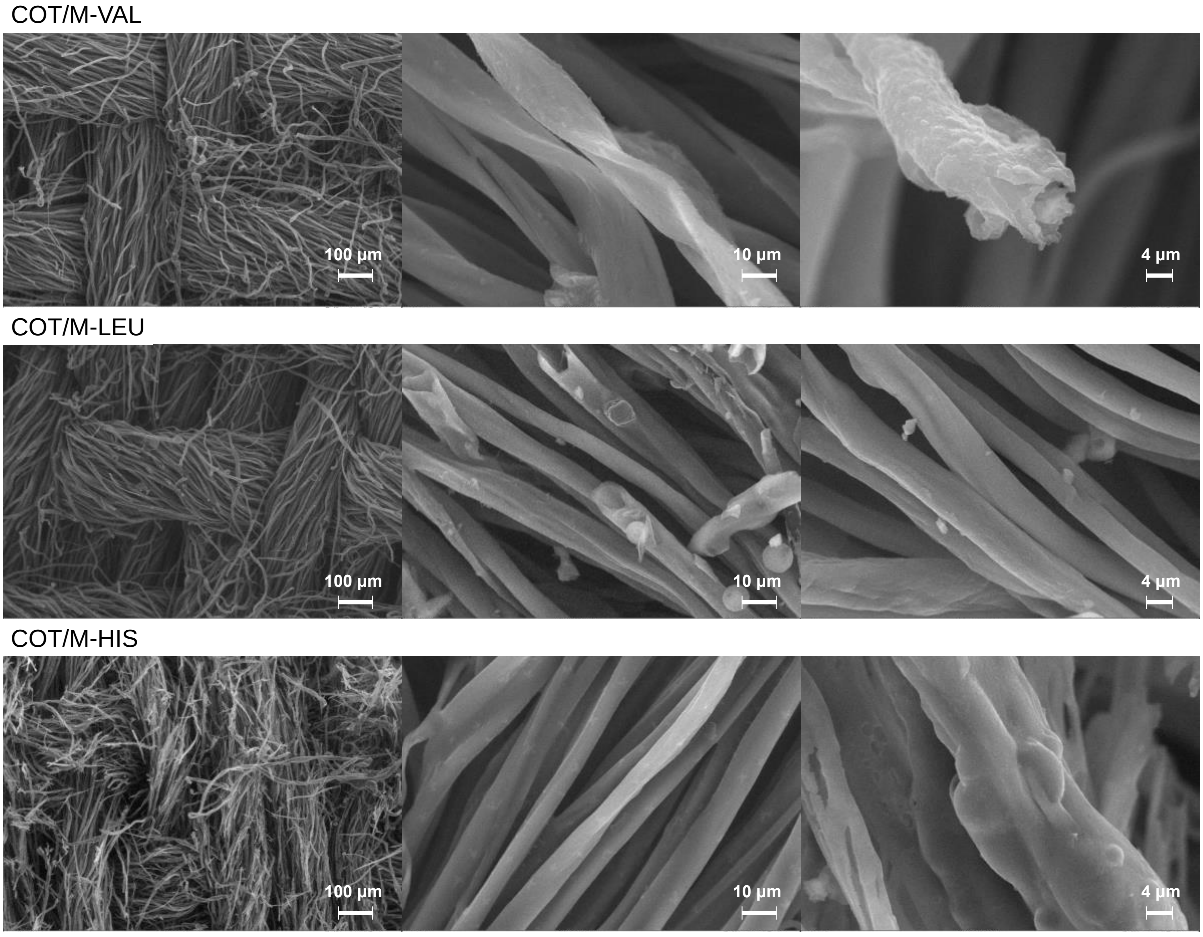

Figure S15. SEM micrographs of COT/MBA-VAL, COT/MBA-LEU and COT/MBA-HIS (add-on: 7%) residues deriving from horizontal flame spread tests.

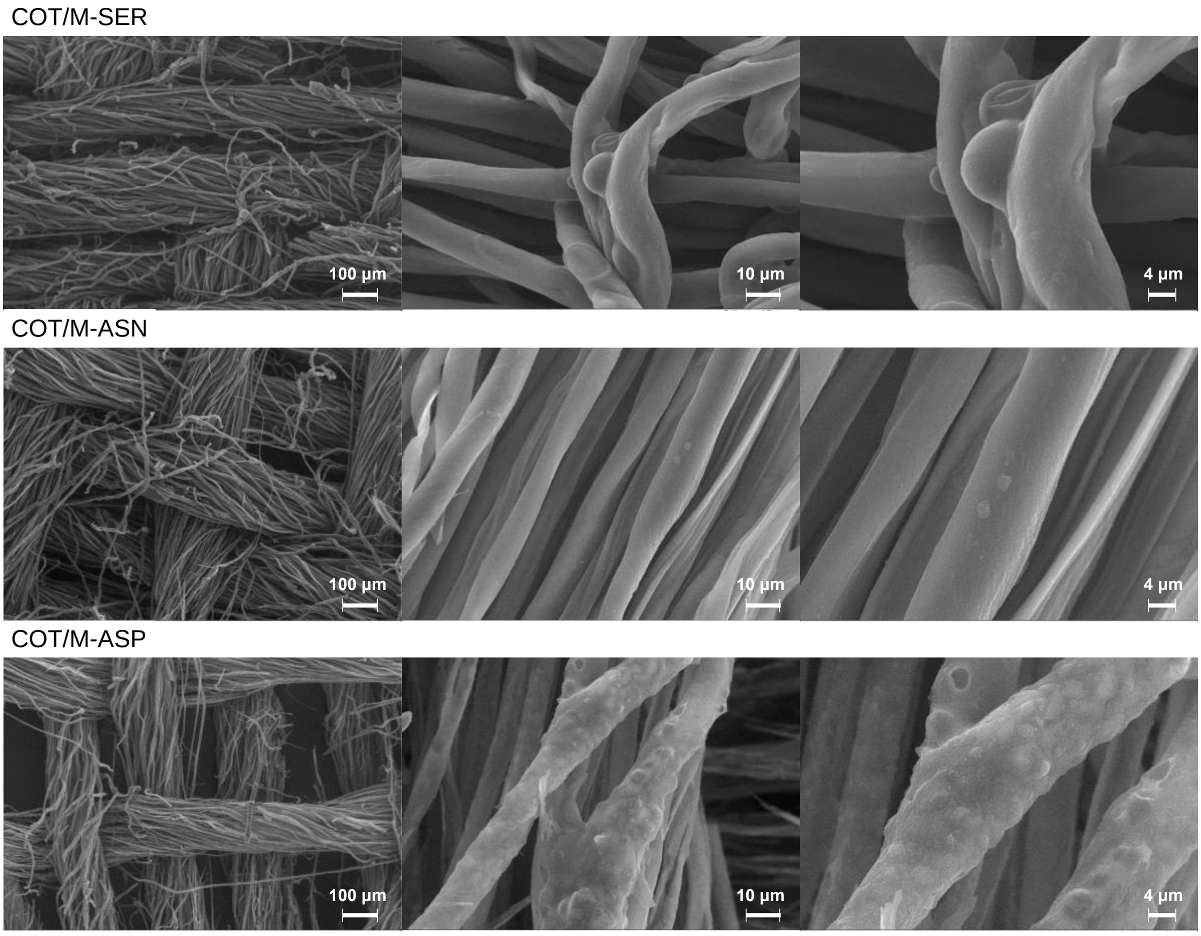

Figure S16. SEM micrographs of COT/MBA-SER, COT/MBA-ASN and COT/MBA-ASP (add-on: 7%) residues deriving from horizontal flame spread tests.
